# The Maimonides Portrait: An Appraisal of One of the World’s Most Famous Pictures

**DOI:** 10.5041/RMMJ.10052

**Published:** 2011-07-31

**Authors:** Yitzhack Schwartz

**Affiliations:** Pediatric Cardiology and Structural Heart Disease, Rambam Health Care Campus, Meyer Children’s Hospital, Haifa, Israel

**Keywords:** Maimonides, portrait, iconography, art, history

## Abstract

Surprisingly, an utterly imaginative “portrait” has become synonymous with Maimonides forever. How and when did this particular portrait become associated with Maimonides? This and many other intriguing questions regarding this portrait are systematically addressed, and its origins, possible inspiration, and hidden objectives are revealed.

The Maimonides portrait is undoubtedly one of the world’s most famous and easily recognizable universal icons. Portraits, including those of Jewish prominent leaders and scholars, became fashionable long after Maimonides died. We have no way of knowing what Maimonides really looked like, yet a single utterly imaginative “portrait” has successfully defined our conception of Maimonides for ever.

Of the numerous available versions of this portrait let us focus on the pen-and-ink drawing frequently cited and known as “portrait and autograph” (Figure 1).^1^ The depicted Maimonides signature in this picture is unequivocally authentic and resembles his numerous verified signatures found in the Cairo Genizah (Figure 2).^2^ However, many intriguing questions come to mind when appraising the portrait itself. In the following article we’ll try to answer these questions.

**Figure 1:**
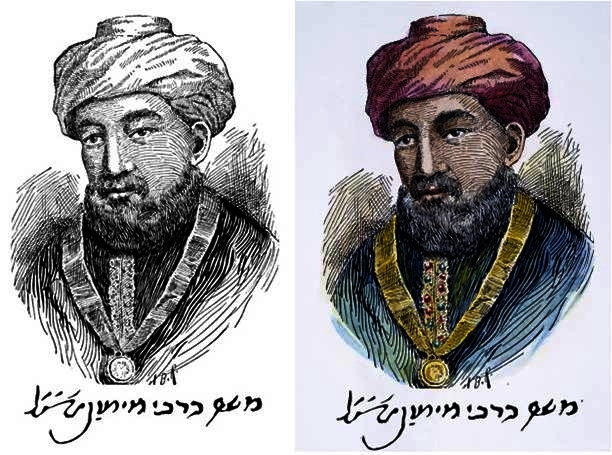
Maimonides’ traditional portrait and autograph.^1^ This nineteenth-century imaginative depiction, courtesy of the Granger Collection, NY, is possibly by the American illustrator Arthur Burdett Frost.

**Figure 2:**
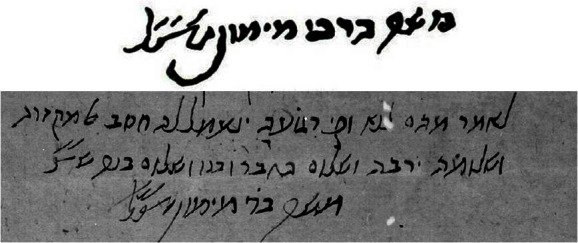
The enlarged signature in the picture (above) compared to the almost identical authentic one found in the Cairo Genizah (below).^2^

## HOW AND WHEN DID THIS PARTICULAR PORTRAIT BECOME ASSOCIATED WITH MAIMONIDES?

The earliest Maimonides portrait, dating back to the fifteenth century, is attributed to Professor Moshe-David (Umberto) Cassuto (1883–1951) who reportedly 3 discovered it in 1935.

Professor Umberto Cassuto, a member of the Academic Council of the Hebrew University in Jerusalem, has discovered a new portrait of Maimonides made in the 15^th^ century. The portrait is coloured and is of rare artistic value, showing Maimonides in oriental dress.

Regretfully, the exact details of that particular intriguing discovery are unknown. Professor Cassuto, a renowned Rabbi and scholar, has written the Maimonides article in the *Treccani Encyclopedia* and was intimately familiar with the rare handwritten and beautifully illuminated copies of the Mishneh Torah created in Italy and Spain in the fifteenth century. It is plausible that, while cataloguing all Hebrew manuscripts in the Vatican Library (later to be published as *Codices Vaticani Hebraici*), Professor Cassuto has indeed encountered and identified such a portrait.

Luckily much more is known about a portrait that dates back to the eighteenth century. This image was probably first “discovered” in the mid-nineteenth century by Yashar (R. Isacco Samuele Reggio, 1784–1855), an Austro-Italian scholar, mathematician, voluminous writer, and rabbi born at Gorizia. Reggio was one of the prominent leaders of Jewish emancipation and found the portrait in a 34-volume encyclopedic work called *Thesaurus Antiquitatum Sacrarum* (1744–1769). Published by Blaseus Ugolinus in Venice, it was written in Latin and (according to its translated title and intent) is a thesaurus of sacred antiquities in which are illustrated the customs, laws and institutions, sacred and civil rites of the ancient Hebrews.

Ugolinus, a Jew by birth, was a very reputable Roman Catholic Christian antiquarian. In this remarkable work Ugolinus did not only bring together reprints of most of the seventeenth-century treatises on Jewish antiquities but also obtained fresh contributors. Moreover he has translated himself numerous treatises as well as extensive parts of the Mishneh Torah, considered Maimonides’ magnum opus.

**Figure 3:**
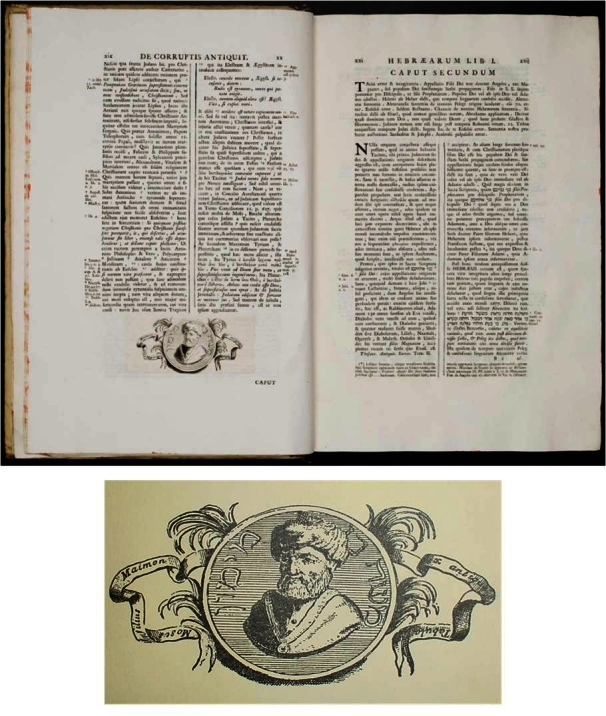
The Maimonides portrait in *Thesaurus Antiquitatum Sacrarum.*^4^ Courtesy of Beinecke Rare Book and Manuscript Library, Yale University.

This modest and somewhat unimpressive miniature portrait (Figure 3)^4^ is considered by most experts, including Professor Richard I. Cohen,^5^ the origin of the Maimonides portrait as we know it.

Moses Margoliouth (1815–1881), a Jewish Christian missionary living in England, sheds important light on the origins of the portrait in the following letter,^6^ dated December 17, 1846:

I know you [i.e. Rev. Dr. J. Horlock] are a profound admirer of that Hebrew sage. I think you will be pleased, therefore, with the accompanying miniature portrait of him. You may have seen it before, for I printed it as a heading to the prospectus of the Philo-Hebraic Society. If not, here it is. I do not think that anybody in England ever saw it before I introduced it. You will, however, wonder whether it is a real likeness, or merely a fictitious one. I will, therefore, give you all the information I possess about it, and judge for yourself.

The famous Italian-Hebrew Scholar, Reggio, discovered it first in that masterpiece of a work, “*Thesaurus Antiquitatum*”, published at Venice by Blaseus Ugolinus. He sent a sketch of his discovery to his friend, Herr Solomon Stern of Berlin. The latter was naturally anxious to know whether the representation was real or imaginary.

Reggio, therefore, sent the following explanation; “In the celebrated work, ‘*Thesaurus Antiquitatum Sacrarum Blasie Ugolini, Venetiis*’, 1744, in the first volume, p. 384, is found the likeness of Maimonides, which the author [of ‘*Thesaurus Antiquitatum*’] says was taken, ‘ex-antiqua tabula’, without, however, stating more fully and circumstantially how he came to the possession of this tabula, where it existed, and if anyone bore testimony to the authenticity of the likeness. However, as Ugolinus is known as an industrious, honorable man, acquainted with his subject, and who cannot easily be suspected of fraud, there is nothing against assuming the probability that at the publication of his work he had really before him such a tabula.”

**Figure 4:**
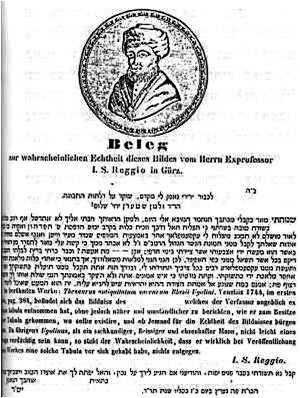
Reggio’s letter to Solomon Stern, December 20, 1843. Note the main text in this correspondence was written in Hebrew whereas when addressing the delicate issue of the portrait’s authenticity Reggio reverted to German.

Herr Solomon Stern printed on one sheet of paper a few copies of the above miniature, accompanied by a copy of Reggio’s letter (Figure 4). I was fortunate enough to get a copy of that document, sent to me by a kind friend from Berlin, who knows my partiality for such literary curiosities.

**Figure 5:**
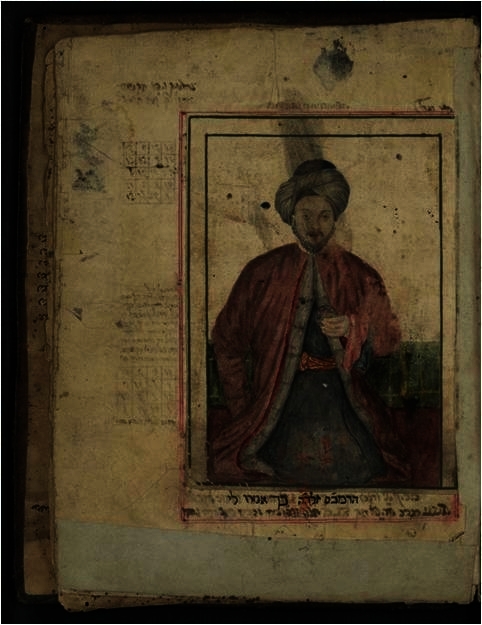
Maimonides portrait.^7^ Maimonides appears to be holding *Nautilus* as a symbol of his broad knowledge of natural sciences. Courtesy of the National Library of Israel, Hebrew University, Jerusalem.

Another hardly known but very interesting eighteenth-century portrait, supposedly from 1769, has somehow reached the Jewish National and University Library in Jerusalem (Figure 5).^7^ Maimonides here appears much younger, and a handwritten puzzling sentence underneath reads (in Hebrew): “Maimonides, may his soul rest in peace in heaven, so I was told”.

**Figure 6:**
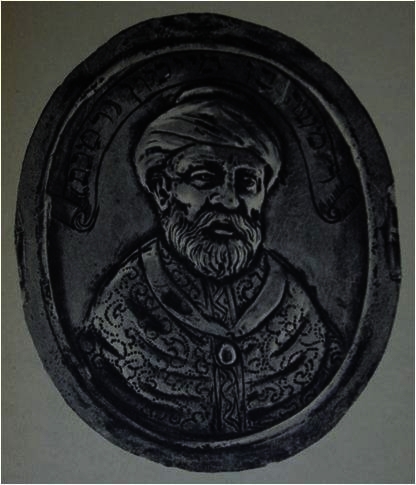
Maimonides bronze medallion from the Renaissance (no exact dating).^8^

The portrait we all associate with Maimonides is thus almost certainly from 1744. It originated in the mid-eighteenth century, was reportedly “discovered” in the mid-nineteenth by Reggio, and disseminated from there. Reggio himself was a painter of considerable ability with more than two hundred drawings and paintings including portraits of many Jewish celebrities. His sketches of the portrait were first forwarded to Germany and soon thereafter to England. Moses Margoliouth “brought” the portrait to England and apparently played an active role in its dissemination. The portrait was not limited to paper and reprints but was also copied on medallions (Figure 6).^8^

**Figure 7:**
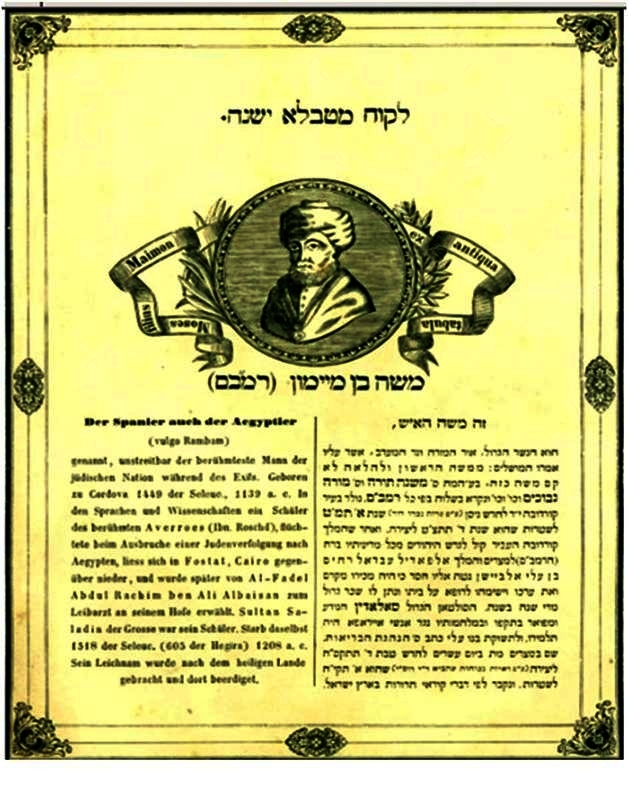
An “authenticity statement” from 1844. Such statements typically accompanied the Maimonides portrait. Note the title in Hebrew that boldly reads “ex-antiqua tabula” and the abbreviated Maimonides biography in both Hebrew and German.

At least some of the circulating portraits were at first accompanied by a rather unusual “authenticity statement” (Figure 7) emphasizing the “ex-antiqua tabula” emblem in Hebrew as an undisputed fact. While the portrait was not common in England in 1847 it was already widespread worldwide by the early twentieth century.

## HOW AUTHENTIC, IF AT ALL, IS THE PORTRAIT?

Ugolinus, the editor of the *Thesaurus Antiquitatum Sacrarum*, claimed that it was copied from an older image engraved or drawn “ex-antiqua tabula”. Although Reggio was convinced of its authenticity, or at least was willing to declare so openly, he refrained from addressing a troubling and fundamental question of whether Maimonides would have approved at all a drawing of his portrait.

According to the particular Jewish religious rules (Halacha) as summarized by Maimonides himself in his seminal work Mishneh Torah (a code of Jewish law):^9^

It is prohibited to make images for decorative purposes, even though they do not represent false deities, as [implied by Exodus 20:23]: “Do not make with Me [gods of silver and gods of gold].” This refers even to images of gold and silver which are intended only for decorative purposes, lest others err and view them as deities.

It is forbidden to make decorative images of the human form alone. Therefore, it is forbidden to make human images with wood, cement, or stone. This [prohibition] applies when the image is protruding – for example, images and sculptures made in a hallway and the like. A person who makes such an image is [liable for] lashes.

In contrast, it is permitted to make human images that are engraved or painted – e.g. portraits, whether on wood or on stone tablets – or that are part of a tapestry.

[The following rules apply regarding] a signet ring which bears a human image: If the image is protruding, it is forbidden to wear it, but it is permitted to use it as a seal. If the image is an impression, it is permitted to wear it, but it is forbidden to use it as a seal, because it will create an image which protrudes.

Similarly, it is forbidden to make an image of the sun, the moon, the stars, the constellations, or the angels, as [implied by Exodus, ibid.]: “Do not make with Me [gods of silver ...]” – i.e. do not make images of My servants, those who serve before Me on high. This [prohibition] applies even [to pictures] on tablets.

The images of animals and other living beings – with the exception of men – and similarly, the images of trees, grasses, and the like may be fashioned. This applies even to images which protrude.

Apparently, since it is religiously permitted, it is very tempting, albeit speculative, to think an artist or one of his students drew or engraved the Maimonides portrait on stone. Nevertheless, it is highly inconceivable that Maimonides would have approved this in his life. Did someone draw his portrait from memory after Maimonides had passed away in 1204 in Egypt and had been buried in Tiberias? Did that hypothetical “portrait” on tablet surface several hundreds of years later far away in Europe, and was it copied and distributed by Ugolinus? I personally find it hard to believe and leave these as open questions to the readers.

It is much more likely that Ugolinus, who was well acquainted with Jewish Halacha in general and Maimonides’ writings in particular, has attached the “ex-antiqua tabula” remark purposefully. Ugolinus knew engraving a portrait on stone is permitted, and he strove to substantiate the authenticity of the portrait and mitigate anticipated Jewish critiques. The ones later engaged in disseminating the portrait have indeed accordingly cited the very same phrase in Hebrew in the title of their authenticity statement (Figure 7).

## ARE THERE EXAMPLES OF SIMILARLY ARCHETYPICAL DRAWINGS THAT PRECEDED AND COULD HAVE SERVED AS INSPIRATION TO THE 1744 MAIMONIDES PORTRAIT?

**Figure 8:**
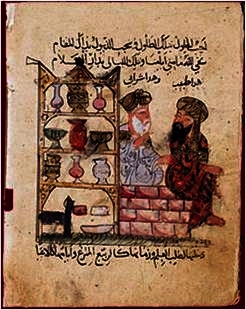
Detail from a miniature from Ibn Butlan’s *Risalat da’wat al-atibba*. Courtesy of L.A. Mayer Museum for Islamic Art, Jerusalem. Photo by Daniela Golan.

In 1058, Ibn Butlan, the famous Egyptian physician and theologist from Bagdad, published a satiric piece of prose (*Risalat da’wat al-atibba*) referring to a young impostor who works as a physician alongside an old and experienced physician who easily exposes his ignorance. The scene (Figure 8), captured in watercolor on papyrus, nicely depicts this elderly respectful physician and the youngster.

This particular picture must have reached early modern Europe along with countless other texts of Middle-Eastern origin that effectively spread the Arabic culture influence. It is quite obvious that such publications might have carved the stereotype of how an old wise physician must have looked.

**Figure 9:**
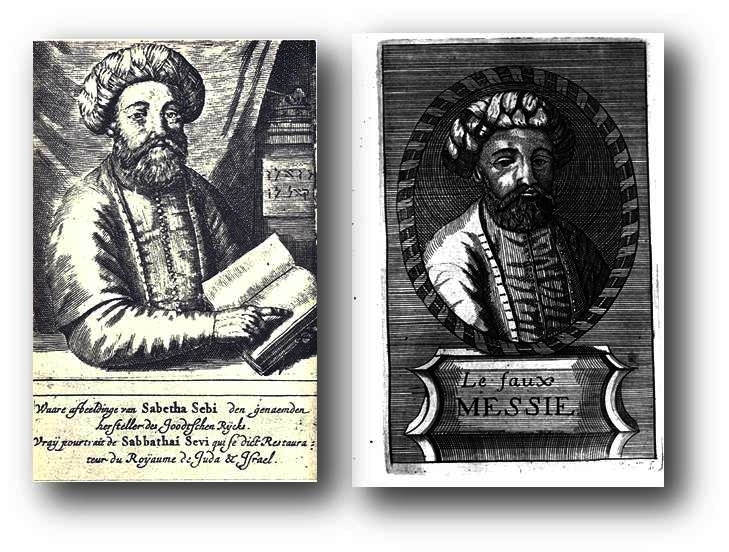
Portraits of “Sabetha Sebi”: from 1669 (left)^10^ and 1728 (right).^11^

In 1669, and again in 1728, two similar portraits of Sabethai Zvi (1626–1676), the Jewish mystic who proclaimed himself Messiah in 1648, were published (Figure 9). These portraits were supposedly made by an eye witness and were thus regarded by many as authentic.^10^,^11^ Whoever drew the Maimonides portrait in 1744 must have been aware of and possibly inspired by these.

## WHO WAS THE ARTIST WHO DREW THE PORTRAIT?

The artist’s identity is regretfully unknown. Ugolinus may have drawn it himself or hired a professional illustrator for the mission.

**Figure 10:**
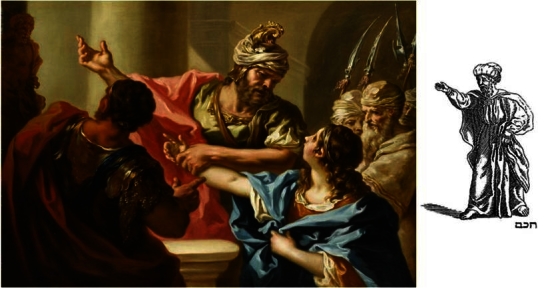
Giovanni Pellegrini (1675–1741): “Young Hannibal Swears Enmity to Rome”, 1731. There is an exceptional intentional resemblance between Hannibal and the Wise Son (or Scholar) of the Amsterdam Haggada (right).

Given the iconographic style similarity between the Maimonides portrait and the Wise Son as depicted in the famous illustration of the Four Sons (Figure 10), dated 1712, it seems plausible that the artists shared some common influences.

Abraham ben Jacob, a convert to Judaism who illustrated the Amsterdam Haggada considered a milestone in the history of Hebrew printing, borrowed most of the illustrations from Mathaeus Merian, a Christian artist. Merian (1593–1650) produced a large number of popular engraved illustrations both for Bibles and history books between 1625 and 1630. It was from among these engravings that the illustrations for the Amsterdam Haggada were chosen. The Wise Son is in fact Hannibal as engraved by Merian in a history book 12. It resembles even better Hannibal as drawn by the Venetian artist Giovanni Pellegrini (1675–1741) in 1731 (Figure 10).

Apparently, Jewish readers in eighteenth-century Europe fully grasped the subtle intentions of the illustrator and indeed associated utmost wisdom with the world-renowned iconic tactician Hannibal just as twentieth-century readers would have associated an image of Albert Einstein with immense genius.

The popularity of the illustrated Haggada with the Jews of Europe was enormous, and accordingly it was copied and recopied in succeeding haggadot printed in Europe and later in the United States well into the twentieth century.

Whoever drew the Maimonides portrait used skillfully the same successful principles of iconographic illustrations incorporating all Maimonides’ characteristics that would have been expected by the target readers.

## DID THE MAIMONIDES WEAR SIDE CURLS (*PE-OT* IN HEBREW), AND WHY DID THE ARTIST DRAW HIM WITHOUT SIDE CURLS AND WITH A TRIMMED BEARD?

According to traditional Jewish rules there are strict prohibitions related to facial hair shaving. Maimonides himself addresses these Jewish rules:^13^

We may not shave the corners of our heads as the idolaters and their priests do, as Leviticus [19:27] states: “Do not cut off the corners of your heads.”

One may remove [the hairs from] the corners [of our heads] with scissors. The prohibition applies only to total removal with a razor.

Did King David have visible Pe-ot as we now know them? Did the rabbis of the Mishna? Did Maimonides? The evidence is wholly against it. Maimonides had most probably kept his side curls mildly trimmed by scissors rather than fully shaved as depicted in the portrait.

Full-grown Pe-ot originated only in the sixteenth century when it became socially important for observant Jews effectively to distinguish Jews from non-Jews and, more importantly, Jews from other Jews who were thought to be too modern.

Whoever drew the Maimonides portrait was not aware that completely shaven side curls and a trimmed beard are in fact characteristic of Islamic figures. Alternatively, the artist may have assumed that because of his high ranking and frequent encounters with kings and ministers Maimonides must have worn clothes that resembled Islamic clothes as well as shaved and trimmed his facial hair like distinguished Islamic figures. Interestingly, Maimonides has indeed allowed this in rare circumstances like his:^14^

A Jew who has an important position in a gentile kingdom and must sit before their kings, and would be embarrassed if he did not resemble them, is granted permission to wear clothes which resemble theirs and shave the hair on his face as they do.

The artist may have even deliberately drawn Maimonides to appear as a Muslim scholar in order to appeal to non-Jewish audiences and emphasize the influence of Islamic culture, science, and medicine on Maimonides rather than his Jewish origin and education.

Key figures engaged in circulating the Maimonides portrait in Europe in the eighteenth and nineteenth centuries were mostly leaders of the Jewish Enlightenment movement (*Haskalah* in Hebrew). *Haskalah* advocated adopting enlightenment values, pressing for better integration into European society, and increasing education in secular studies, Hebrew language, and Jewish history. *Haskalah* in this sense marked the beginning of the wider engagement of European Jews with the secular world, ultimately resulting in the first Jewish political movements and the struggle for Jewish emancipation. Maimonides’ portrait lacking characteristic Jewish features was in line with their motto “Be a Jew at home, and a man in the street”.

**Figure 11:**
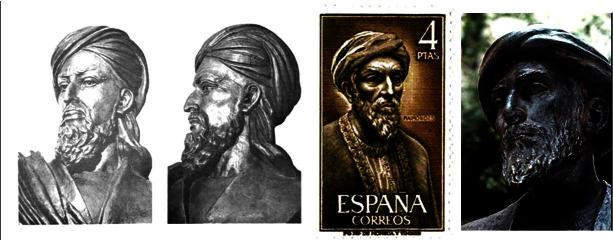
Maimonides statue (A), erected in 1964 in Tiberiadus Square, Juderia (Jewish old quarter), Cordoba and depicted on a Spanish stamp issued 1967 (B). The Ibn Sina (Avicenna, *c.* 980–1037)) statue (C,D) (http://www.muslimphilosophy.com) may have served as inspiration.

It is of interest to note that, unlike in the portrait, in the renowned statue in Cordoba (Figure 11) Maimonides does appear to have side curls. This may reflect the artist’s perception of prominent, especially religious, Jews.

However, the sculptured face bears remarkable resemblance to the great physician and philosopher Ibn Sina (Avicenna, 980–1037) (Figure 11) whose sculpture was based on an accurate reconstruction of his skull. Evidently, all artists look for inspiration and historical, tangible references and often resort to using archetypical faces collectively believed to characterize distinguished ancient scholars/physicians/philosophers not necessarily Jews.

## WHAT IS THE SIGNIFICANCE OF HIS UNIQUE TURBAN AND RICHLY DECORATED CLOTHES?

Whether the artist has chosen a garment and decorations based on historical factoids or was simply influenced by stereotypes and existing living models is undetermined. The end result might be coincidental, but some symbolic hints warrant further reflection.

Egyptians and Orientals, including local Jews, have indeed worn typical turbans for many centuries. A typical turban known as the Moock’leh resembles the one in the Maimonides portrait.^15^

In certain periods of history, different colored turbans were mandated by law according to one’s religion. There were also identifiable differences in the manner of wrapping them. Nicolas de Nicolay who returned from Istanbul in 1552 reported that Jewish turbans were yellow/orange, a color that matches the one in the Maimonides portrait. His published original engravings and observations of the Orient^16^ include a famous impression of a Merchant Jew considered a trustworthy representation of the turban and clothes worn by rich eminent Jews.

The decorated clothes may also allude to the usage of amulets or talismans. Maimonides himself disrespected and even preached strongly against the protective and healing powers of amulets or blessed objects. Nevertheless at the time when the Maimonides portrait was published most people of all religions, including Jews, believed in those powers, and the artist may have drawn the “medal” or engraved coin accordingly.

Alternatively, this may simply represent a generally accepted trade-mark of ancient physicians, almost like wearing a stethoscope nowadays is considered a trade-mark of modern physicians. Furthermore the artist may have alluded to an honorary ranking symbol given to Maimonides as head physician of Salah-a-Din, the Sultan of Egypt.

The embroidery and what appears like gold braid with 12 colored gemstones might even be the artist’s interpretation of the Hoshen, the sacred breastplate worn by the High Priest for the Israelites. In the biblical account, the breastplate is termed the *breastplate of judgment*, because the Urim and Thummim (four rows of three engraved gems), which were used in divination, were placed within it.

There is a close, yet probably coincidental, resemblance between the general outline of the decorations and the Temple Menorah drawn by Maimonides himself.^17^ It was quite fashionable in the sixteenth and seventeenth century to incorporate hidden, barely noticeable, hints or secret messages in works of art. One can only wonder whether the anonymous artist tried cleverly to increase the credibility and authentic value of the portrait by using this particular outline that many educated Jewish readers would subconsciously recognize.

Interestingly, ever since the beginning of the seventeenth century the Sephardic Chief Rabbi, the *Rishon LeZion* (“First to Zion” in Hebrew), wears gold-braided robe and turban. The outfit is partially reminiscent of the Hakham Bashi who used to represent the Jews in front of the Turkish Ottoman authorities, but in its current format it is majorly in the wake of Maimonides.

In conclusion, it would seem that the early Hebrew printers in Venice, eager to supply their readers with a tangible likeness of Maimonides, had simply pulled out an available piece of “clip art” that conveyed a rough image of what an Egyptian Jewish scholar might have looked like. The original unknown artist has very successfully portrayed a remarkably convincing image that has captivated the imagination and become synonymous with Maimonides ever since.

The artist and/or the one who ordered the portrait must have been Jews by birth with in-depth understanding of Jewish Halacha and Maimonides’ rulings. This specific Maimonides portrait was distributed by supporters of the Jewish enlightenment movement and served their objectives.

**Figure 12:**
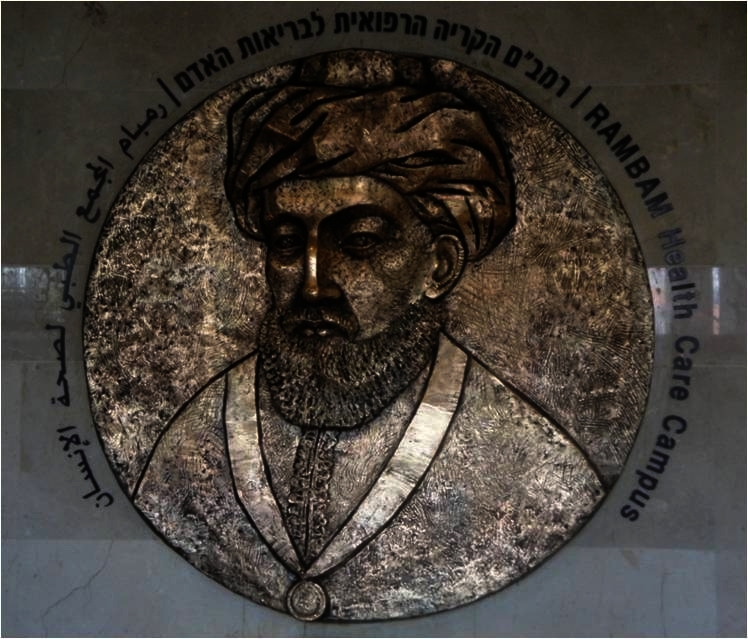
Plaque of Maimonides at the main entrance hall, Rambam Medical Center, Haifa, Israel. Photo by Pioter Fliter.

It is impossible to count the number of times this portrait has been cited and the number of artists who have used it as an inspiration to their work. It is only appropriate that the Maimonides plaque based on this famous portrait proudly hangs at the main entrance to the Rambam Health Care Campus, Haifa, named after Maimonides (Figure 12).

## References

[1] The Granger CollectionNY: 0004896_T and 0046323_T.

[2] Cairo Genizah Collection.

[3] The Palestine Post, August 20, 1935, from Palestine Telegraphy Agency.

[4] Beinecke Rare Book and Manuscript Library, Yale University: Bibliographic Record Number 2008140, Image ID Number 1041737.

[5] Cohen RI (1998). Jewish Icons: Art and Society in Modern Europe.

[6] Margoliouth M (1850). Pilgrimage to the land of my fathers. Letter XIV.

[8] Wurmbrand M Roth C Le Peuple Juif, quatre mille ans de survivance ParisAlbin Michel1967 116

[9] Maimonides M, Mishneh Torah, Madda Sefer, Zarah Avoda

[10] Thomas Coenen (1669). Ydele verwachtinge der Joden getoont in den persoon van Sabethai Zevi.

[11] Jean Baptiste de Rocoles (1728). Les Imposteurs Insignes.

[12] Merian M (1657). Hannibal the General of Carthage Swears to Conquer Rome. J. L. Gottfried’s Historische Chronica.

[13] Maimonides M, Mishneh Torah, Sefer Madda, Avoda Zarah

[14] Maimonides M, Mishneh Torah, Sefer Madda, Avoda Zarah

[15] Lane EW (1836). An Account of the Manners and Customs of the Modern Egyptians.

[16] Nicolas de Nicolay Les Quatre Premiers Livres des navigations et peregrinations orientales.

[17] (1967). Maimonides illustrated comment. Perush Hamishnayot, Menachot 3:7.

